# Smoking cessation and incident dementia in elderly Japanese: the Ohsaki Cohort 2006 Study

**DOI:** 10.1007/s10654-020-00612-9

**Published:** 2020-02-15

**Authors:** Yukai Lu, Yumi Sugawara, Shu Zhang, Yasutake Tomata, Ichiro Tsuji

**Affiliations:** Division of Epidemiology, Department of Health Informatics and Public Health, Tohoku University School of Public Health, Graduate School of Medicine, 2-1 Seiryo-machi, Aoba-ku, Sendai, Miyagi 980-8575 Japan

**Keywords:** Smoking, Smoking cessation, Incident dementia, Cohort study, Elderly population

## Abstract

To investigate the association of smoking status and years since smoking cessation with the risk of incident dementia among elderly Japanese. We conducted a longitudinal analysis of smoking status and smoking cessation with dementia in prospective cohort study of 12,489 Japanese individuals aged ≥ 65 years who were followed up for 5.7 years. Information on smoking status and other lifestyle factors was collected via a questionnaire in 2006. Data on incident dementia were retrieved from the public Long-term Care Insurance Database. The Cox proportional hazards model was used to estimate the multivariable-adjusted hazard ratios (HRs) and 95% confidence intervals (95% CIs) for incident dementia. During 61,613 person-years of follow-up, 1110 cases (8.9%) of incident dementia were documented. Compared with individuals who had never smoked, current smokers showed a higher risk of dementia (HR 1.46, 95% CI 1.17, 1.80). Among ex-smokers, the risk for those who had stopped smoking for ≤ 2 years was still high (HR 1.39, 95% CI 0.96, 2.01), however, quitting smoking for 3 years or longer mitigated the increased risk incurred by smokers; the multivariable HRs (95% CIs) were 1.03 (0.70, 1.53) for those who had stopped smoking for 3–5 years, 1.04 (0.74, 1.45) for 6–10 years, 1.19 (0.84, 1.69) for 11–15 years, and 0.92 (0.73, 1.15) for > 15 years. Our study suggests that the risk of incident dementia among ex-smokers becomes the same level as that of never smokers if they maintain abstinence from smoking for at least 3 years.

**Electronic supplementary material:**

The online version of this article (10.1007/s10654-020-00612-9) contains supplementary material, which is available to authorized users.

## Introduction

Dementia is a major cause of disability and dependency among the elderly, having a significant impact on individuals as well as families, communities and societies. In 2015, dementia affected about 47 million people worldwide, and it is estimated that globally nearly 9.9 million people develop dementia each year [1]. Thus, it is critical to identify more modifiable factors to reduce the incidence of dementia.

The association between smoking status and dementia has been examined extensively [2–12], and a recent meta-analysis indicated that current smokers have a significantly higher risk of dementia [13]. However, it has been suggested that smoking cessation would attenuate the excess risk of dementia [13], in addition to other diseases such as cardiovascular diseases (CVDs) [14–17], cancers [18], and chronic obstructive pulmonary disease (COPD) [19]. Previous studies have indicated the risk of CVDs among smokers starts to decline within 2–4 years after smoking cessation [14–16]. On the other hand, it appears that over 10 years of smoking cessation would be necessary until the risk of cancer began to decline [18]. However, it is still uncertain how long a period of smoking cessation would begin to reduce the risk of dementia. One study has compared the risk of all-cause dementia, Alzheimer’s disease (AD), and vascular dementia (VaD) between current smokers and ex-smokers, and found that the risk decreased in those who had quit for 4 years or longer [20].

The present cohort study examined the association of smoking status and smoking cessation with incident dementia, focusing particularly on how long a period of abstinence would be necessary in order for the risk of dementia among ex-smokers to reach the same level as that in never smokers.

## Methods

### Study cohort

The design of the Ohsaki Cohort 2006 Study has been described in detail elsewhere [21]. In brief, the source population for the baseline survey comprised 31,694 men and women aged ≥ 65 years who were living both at home and in long-term care settings, in Ohsaki City, north-eastern Japan, on 1 December 2006.

The baseline survey was conducted between 1 December and 15 December 2006, and follow-up of the participants was started from 1 April 2007. A questionnaire was distributed by the heads of individual administrative districts to individual households and then collected by mail. In this analysis, 23,091 persons who provided valid responses formed the study cohort. We excluded 6333 people who did not provide written consent for review of their long-term Care Insurance (LTCI) information, 2102 who had already been certified as having disability by the LTCI before follow-up (1 April 2007), 62 who had died or moved out of the district before follow-up, 192 whose Doctor’s Opinion Paper (DOP) or cognitive status in the DOP were unavailable, 1828 whose data on smoking status were missing, and 85 whose data on years since smoking cessation were missing. Thus, 12,489 participants were finally included in the analysis for the purposes of this study.

### Smoking status

Participants were categorized as never smokers, current smokers, or ex-smokers according to their responses on the questionnaires at the baseline. Both current smokers and ex-smokers were asked when they had started smoking, and the average number of cigarettes smoked per day; ex-smokers were also asked the number of years that had passed since they had quit. We initially intended to categorize ex-smokers into “≤ 5 years”, “6–10 years”, “11–15 years”, and “> 15 years”, but previous studies have indicated that an even shorter period of smoking cessation can benefit cognitive function [20, 22]. Thus, we decided to further divide “≤ 5 years” into two separate groups, “≤ 2 years” and “3–5 years”. Additionally, we also calculated cumulative smoking pack-years (the average number of cigarettes smoked per day divided by 20 and multiplied by the number of years of smoking) among both current smokers and ex-smokers, and considered never smokers as 0 pack-years.

### Follow-up (incident dementia)

The primary outcome was incident dementia, defined as disabling dementia according to the criteria of the LTCI system that has been implemented in Japan since April 2000 [23]. The LTCI is a mandatory form of national social insurance to assist activities of daily living (ADLs) in the disabled elderly [24–26]. Everyone aged 40 years or older pays premiums and everyone aged 65 years or older is eligible for formal caregiving services under a uniform standard of disability certification. The procedure for disability certification comprises two parts: assessment of the degree of functional disability using a questionnaire developed by the Ministry of Health, Labour and Welfare, and reference to the DOP prepared by the attending physician [27]. The DOP is a standard form used for assessing patients’ chronic medical conditions and functions of daily life.

Disabling dementia was defined as incident functional disability with dementia according to the LTCI system, whereby the dementia exceeded rank I (≥ rank II) on the Dementia Scale (Degree of Independence in Daily Living for Elderly with Dementia), as entered on the DOP. The Dementia Scale is classified into six ranks: 0, I–IV, M; Rank M means that an individual has severe dementia-related behavioural disturbance that requires medical intervention, and a rank exceeding I is typically used as an outcome measure of incident dementia because individuals who have mild or moderate dementia are classified as rank II [23, 28–30]. A previous study reported that the Dementia Scale had a sensitivity (95% CI) of 73% (65–80%) and a specificity of 96% (94–97%) against clinical diagnoses by neuropsychiatrists who used a clinical interview as defined by the International Psychogeriatric Association [31].

We obtained a dataset that included information on LTCI certification, death or emigration from Ohsaki City. All data were transferred from the Ohsaki City Government under an agreement related to Epidemiologic Research and Privacy Protection.

### Covariates

Body mass index (BMI) was calculated as the self-reported body weight (kg) divided by the square of the self-reported body height (m) and BMI ≥ 25 kg/m^2^ was defined as obesity. Time spent walking was evaluated by asking the question, ‘How long do you walk per day, on average?’, for which the participants chose one of three responses: ‘< 0.5 h’, ‘0.5–1 h’ or ‘≥ 1 h’. Alcohol drinking status was also obtained by asking the question, “Do you drink alcohol?”, for which the participants chose one of three responses: ‘yes’, ‘abstain’ or ‘no’. The participants were also asked about whether they had ever suffered from the following diseases: stroke, myocardial infarction (MI), hypertension (individuals with self-measured systolic blood pressure ≥ 140 mmHg or diastolic blood pressure ≥ 90 mmHg were also defined as hypertensive) or diabetes. Education level was assessed using the question, “How old were you when you left school?” and based on the responses, we further divided the participants into junior high school or less (< 16 years), high school (16–18 years) or college or higher (≥ 19 years). Psychological distress was measured using the Kessler 6-item Psychological Distress Scale [32, 33]. Using six questions, respondents were asked about their mental status over the previous month. Total point scores ranged from 0 to 24. Based on the optimal cut-off point for mental illness in the validation study, we classified individuals with scores of ≥ 13 as having psychological distress [33].

Cognitive function was measured using items from the Kihon Checklist, which was developed by the Ministry of Health, Labour, and Welfare of Japan to predict functional decline in community-dwelling elderly. Respondents were asked about their current subjective memory complaints by using three binary questions yielding total point scores ranging from 0 to 3. The validity of the cognitive function score in the Kihon Checklist had been confirmed in a previous study using the Clinical Dementia Rating as a gold standard [34].

### Ethical issues

Informed consent was obtained from all individual participants included in the study. We considered the return of completed questionnaires to imply consent to participate in the study involving the baseline survey data and subsequent follow-up of death and emigration. We also confirmed information regarding LTCI certification status after obtaining written consent along with the questionnaires returned from subjects at the time of the baseline survey in 2006. The Ethics Committee of Tohoku University Graduate School of Medicine (Sendai, Japan) reviewed and approved the study protocol. (Approval No.2016-1-586).

### Statistical analysis

We counted the person-years of follow-up for each subject from 1 April 2007 until the date of incident dementia, date of emigration from Ohsaki City, date of death, incident functional disability without dementia or the end of the study period (30 November 2012), whichever occurred first.

The multivariable-adjusted Cox proportional hazards model was used to calculate the hazard ratios (HRs) and 95% confidence intervals (95% CIs) for incident dementia according to smoking status. Dummy variables were created for the smoking status groups, and never smokers were defined as a reference category. Time of follow-up was used as the time scale in the corresponding models. Multivariable models were adjusted for the following variables. Model 1 was adjusted for sex and age (continuous). To examine whether the association between smoking status and dementia was attributable to lifestyle factors and other health-related factors, model 2 was further adjusted for obesity, time spent walking per day, alcohol drinking status, education level, history of diseases (stroke, MI, hypertension, or diabetes), and psychological stress score.

Smoking is a significant risk factor for death, and some severe diseases that can cause disability and death without disability, as well as non-dementia disability, were censored during follow-up before the onset of dementia in our study. Accordingly, it is necessary to examine the possible influence of competing events on the association between smoking status and risk of incident dementia applying Fine and Gray’s subdistribution hazards regression model [34]. Competing events were defined as: (1) death without disability, (2) any other types of disability and (3) death and any other types of disability.

Four sets of sensitivity analyses were undertaken. First, considering possible reverse causality, people with higher cognitive function are more likely than those with lower cognitive function to quit smoking, we analyzed whether the association would change if only individuals who had higher cognitive function at the baseline were selected; in this sensitivity analysis, “Cognitive function score in the Kihon Checklist = 0 points” was defined as better cognitive function. Second, we reanalyzed the association between smoking cessation and dementia by excluding participants whose dementia event occurred in the first 2 years of follow-up. Third, considering that smoking prevalence differs according to sex, age, education level and cardiovascular risk factors (CRFs), and that education and CRFs are also important factors related to incident dementia, we stratified participants according to sex, age (< 75 years or ≥ 75 years), education level (< junior higher school or ≥ junior high school), history of stroke or MI (at least one of them), hypertension, and diabetes. Tests of interaction were also performed in terms of sex, age, education level, and history of diseases mentioned above. Lastly, to explore further the continuous relationship between years since smoking cessation and incident risk of dementia, penalized splines (P-splines) were used where automatic selection criteria for deciding the optimal degree of smoothing (or equivalently, the optimal degrees of freedom) were implemented [35].

In addition, previous studies also suggest that cumulative smoking pack-years was related to cognitive function [36]. Therefore, we also applied multivariable Cox models to examine the relationship between cumulative smoking pack-years and incident dementia, with never smokers as the reference group.

All of the above analyses were performed using SAS version 9.4 (SAS Inc.), and P-splines were drawn by *smoothHR* package using R version 3.5.2. All statistical tests described here were two-sided, and differences at *P* < 0.05 were accepted as statistically significant.

## Results

### Characteristics of the subjects

**Fig. 1:**
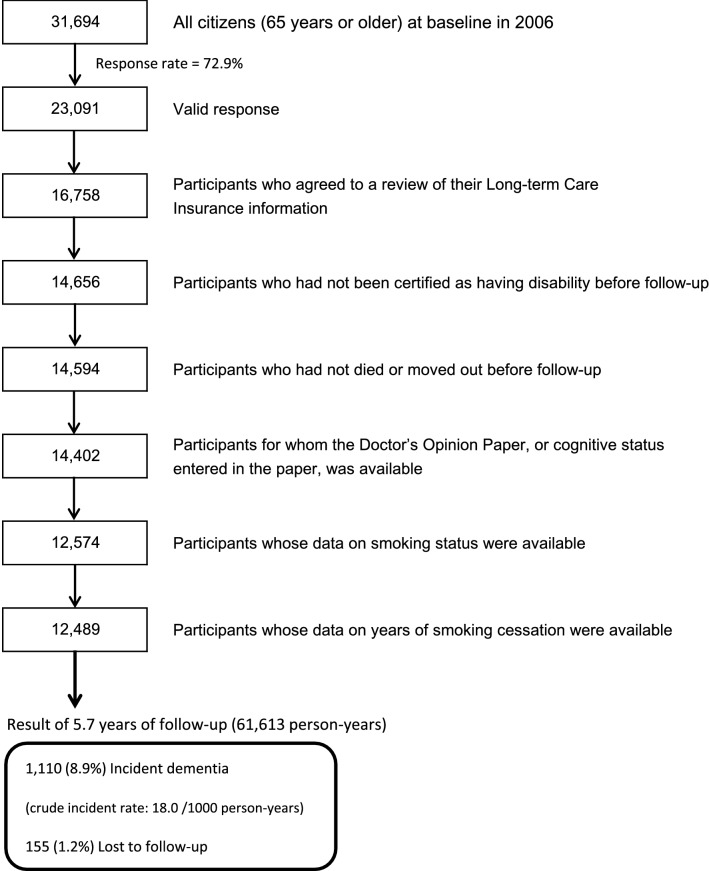
Flow chart of study participants

During the 5.7-year follow-up period, 155 persons were lost to follow-up because of migration from the study area, leaving a follow-up rate of 98.8%. Among a total of 61,613 person-years, 1110 cases of incident dementia were documented, accounting for 8.9% of the total study participants. The crude incident rate for dementia in our study was 18.0 per 1000 person-years (Fig. 1), which was comparable to the other two cohort studies of older Japanese (i.e. 23.3 and 28.0 per 1000 person-years, respectively) [37, 38].

Table 1 shows the baseline characteristics of the study participants according to smoking status; 59.6% of them were never smokers, 13.8% were current smokers, and 26.6% were ex-smokers at the baseline. Never smokers were more likely to be female, to have a higher education level, and to be free of stroke, MI or diabetes, whereas current smokers were more likely to be younger, not obese and current drinkers, and to have a lower education level. Ex-smokers tended to be more likely to have suffered from stroke, MI or diabetes than both current and never smokers.

**Table 1:** Characteristics of participations by smoking status at baseline (N = 12,489)

	Smoking Status							*P* value^a^
	Never smokers (n = 7443, 59.6%)	Current smokers (n = 1718, 13.8%)	Ex-smokers (years since smoking cessation; n = 3328, 26.6%)					
			≤ 2 years (n = 383)	3–5 years (n = 411)	6–10 years (n = 600)	11–15 years (n = 407)	> 15 years (n = 1527)	
Age (years) (mean ± SD)	73.9 ± 6.0	72.3 ± 5.5	72.6 ± 5.1	73.0 ± 5.4	73.3 ± 5.4	74.2 ± 5.5	75.3 ± 6.2	< 0.0001
Male (%)	20.2	88.2	90.3	89.5	92.2	94.4	95.6	< 0.0001
Obesity (%)^b^	32.2	22.1	26.0	30.9	34.4	30.9	30.8	< 0.0001
Education level (junior high school or less) (%)^c^	27.4	34.3	32.2	32.0	31.2	34.3	33.8	< 0.0001
Current drinkers (%)	22.5	64.0	55.9	54.5	58.2	55.8	60.1	< 0.0001
Time spent walking (≤ 0.5 h/day) (%)	35.5	33.9	39.6	36.0	34.1	36.6	32.7	0.21
Psychological stress (%)^d^	4.6	4.3	2.4	3.7	4.1	3.9	4.1	0.54
History of diseases (%)								
Stroke	2.2	3.1	4.4	3.7	4.3	5.2	4.3	< 0.0001
Myocardial infarction	3.5	3.8	7.3	9.3	10.3	9.1	9.0	< 0.0001
Hypertension	56.0	55.1	56.1	52.8	59.8	62.4	62.5	< 0.0001
Diabetes	10.5	12.5	13.6	15.3	16.8	16.7	15.5	< 0.0001

^a^*P* values were calculated by using χ^2^ test for variables of proportion and one-factor ANOVA for continuous variables

^b^Body mass index ≥ 25 kg/m^2^

^c^Age at completion of education < 16 years old

^d^Kessler 6-item Psychological Distress Scale score ≥ 13

### Smoking cessation and incident dementia

**Fig. 2:**
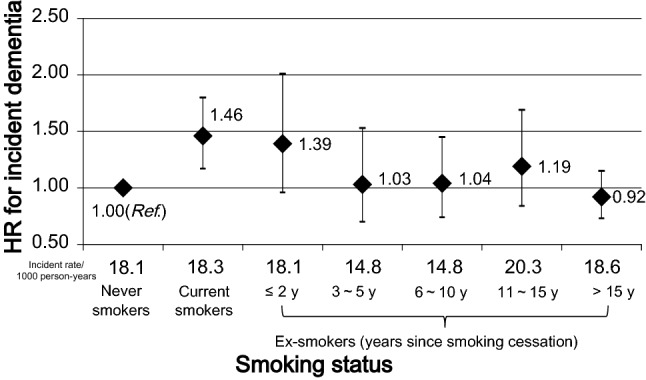
Association between years since smoking cessation and incident risk of dementia. *Note* Hazard ratios were adjusted for sex, age, obesity, time spent walking per day, alcohol drinking status, education level, history of stroke, myocardial infarction, hypertension, or diabetes, and psychological stress score

Table 2 and Fig. 2 show the association between smoking status and incident dementia. Compared with never smokers, current smokers showed an elevated risk of incident dementia; the HR (95% CI) was 1.46 (1.17, 1.80) in the multivariable-adjusted model. On the other hand, among ex-smokers, the multivariable HR (95% CI) was 1.39 (0.96, 2.01) for those who had stopped for ≤ 2 years before the baseline, which still showed a high risk. Furthermore, the elevated risk in current smokers is no longer seen among those who had stopped smoking for 3 years or longer; the multivariable HRs (95% CIs) were 1.03 (0.70, 1.53) for those who had stopped for 3–5 years, 1.04 (0.74, 1.45) for 6–10 years, 1.19 (0.84, 1.69) for 11–15 years, and 0.92 (0.73, 1.15) for > 15 years.

**Table 2:** Association between smoking status and incident dementia (N = 12,489)

Smoking status	No. of participants	No. of dementia cases	Person-years	Incident rate/1000 person-years	Model 1^a^	Model 2
Never smokers	7443	673	37,143	18.1	1.00 (*ref.*)^b^	1.00 (*ref.*)
Current smokers	1718	154	8407	18.3	1.53 (1.24, 1.89)^c^	1.46 (1.17, 1.80)
Ex-smokers (years since smoking cessation)						
≤ 2	383	34	1874	18.1	1.50 (1.04, 2.17)	1.39 (0.96, 2.01)
3–5	411	29	1960	14.8	1.14 (0.77, 1.69)	1.03 (0.70, 1.53)
6–10	600	43	2908	14.8	1.11 (0.80, 1.55)	1.04 (0.74, 1.45)
11–15	407	40	1968	20.3	1.31 (0.93, 1.85)	1.19 (0.84, 1.69)
> 15	1527	137	7353	18.6	0.97 (0.77, 1.21)	0.92 (0.73, 1.15)

^a^Model 1 was adjusted for sex and age (continuous)

^b^Model 2 was adjusted for model 1 plus education level (junior high school or less, high school, college or higher, or missing), obesity (yes, no, or missing), time spent walking (≥ 1 h, 0.5–1 h, < 0.5 h, or missing), alcohol drinking status (never, former, current, or missing), history of diseases (stroke, myocardial infarction, hypertension, or diabetes [yes or no]), and psychological stress score (< 13, ≥ 13, or missing)

^c^Hazard ratios (HRs) and 95% confidence intervals (95% CIs) were calculated by Cox proportional hazards models

Table 3 shows the results of competing-risk models. We considered three different types of competing events; however, the results revealed consistently similar association patterns between years since smoking cessation and incident risk of dementia. In addition, to disentangle the effects attributed to the duration of previous smoking history and duration of smoking cessation, we further adjusted for cumulative smoking pack-years (continuous), but the results remained the same (data not shown).

**Table 3:** Association between smoking status and incident dementia in competing-risk models

Smoking status	No. of dementia cases	Competing-risk model of death		Competing-risk model of other types of disability		Competing-risk model of other types of disability and death	
		No. of competing events	Multivariable model^a^	No. of competing events	Multivariable model	No. of competing events	Multivariable model
Never smokers	673	501	1.00 (*ref.*)^b^	836	1.00 (*ref.*)	1167	1.00 (*ref.*)
Current smokers	154	248	1.35 (1.08, 1.68)	149	1.41 (1.14, 1.75)	338	1.34 (1.08, 1.66)
Ex-smokers (years since smoking cessation)							
≤ 2	34	63	1.34 (0.92, 1.94)	33	1.41 (0.97, 2.05)	82	1.36 (0.94, 1.96)
3–5	29	72	0.94 (0.63, 1.40)	36	1.04 (0.70, 1.55)	93	0.94 (0.63, 1.40)
6–10	43	87	0.95 (0.67, 1.34)	70	0.97 (0.69, 1.37)	129	0.94 (0.66, 1.33)
11–15	40	63	1.18 (0.83, 1.66)	36	1.24 (0.88, 1.74)	82	1.20 (0.85, 1.70)
> 15	137	214	0.89 (0.70, 1.12)	170	0.91 (0.72, 1.14)	313	0.88 (0.70, 1.12)

^a^Multivariable model was fully adjusted for the same covariates as Model 2 in the Table 2

^b^Hazard ratios (HRs) and 95% confidence intervals (95% CIs) were calculated by Fine and Gray’s subdistribution hazards regression model

### Sensitivity analysis

In the sensitivity analysis that excluded dementia cases that had been ascertained in the first 2 years of follow-up, the results did not change substantially. In comparison with never smokers, current smokers and ex-smokers quitting for 2 years or less had a higher risk of dementia, whereas ex-smokers quitting for 3 years and longer still did not show an increased risk of dementia (Table S1). We then reanalyzed our data after excluding participants who had shown poorer cognitive function at the baseline. The risk of dementia was slightly attenuated in the multivariable model among current smokers, whereas for ex-smokers, the pattern of dementia risk remained similar in the age- and sex-adjusted model and the multivariable-adjusted model (Table S1).

**Fig. 3:**
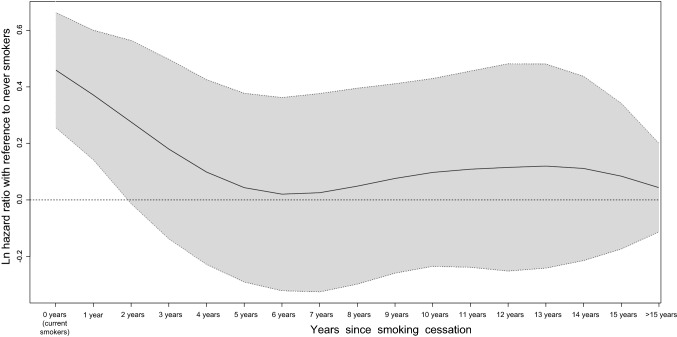
Continuous association between years since smoking cessation and incident risk of dementia with reference to never smokers. *Note* This P-spline curve was adjusted for sex, age, obesity, time spent walking per day, alcohol drinking status, education level, history of stroke, myocardial infarction, hypertension, or diabetes, and psychological stress score. Ln hazard ratio = 0 (horizontal dotted line) is equivalent to hazard ratio = 1.0. Solid line: spline curve of estimated ln hazard ratio; shaded area: 95% confidence interval

Figure 3 shows the continuous association between years since smoking cessation and incident dementia with reference to never smokers. We observed that, among ex-smokers quitting for 3 years or longer, the lower limit of the 95% CI for ln hazard ratio was no longer positive, and further, those quitting for 5 years or longer had a similar level of risk (ln hazard ratio) as never smokers. The nature of the association between smoking cessation and incident dementia was consistent with our main findings (Table 2, Fig. 2).

The results of stratification analyses according to sex, education, history of CVDs, hypertension and diabetes were similar to the main findings, except the results according to age in which current smokers aged 75 or older showed a similar risk compared to never smokers. This issue may be attributed to smokers having died due to other severe diseases caused by smoking before being diagnosed with dementia (Table S2). Further, regarding the stratification analysis according to cumulative smoking pack-years (≤ 20 vs. > 20), the hazard ratios also did not change substantially (Table S3).

## Discussion

In this cohort study of elderly Japanese, we investigated the association between smoking status and the risk of incident dementia and further examined when the increased risk among smokers began to decrease through smoking cessation. Our findings confirmed the results of previous studies [2–12] indicating that smoking was associated with an increased risk of incident dementia, while quitting smoking for 3 years or longer mitigated the increased risk incurred by smokers. Furthermore, our study found that ex-smokers who had quit for ≤ 2 years still showed an increased risk, but that the risk was reduced to a level comparable with that in never smokers if smoking cessation was maintained for at least 3 years.

### Sensitivity analysis

To minimize the effects of reverse causality, we repeated all analyses after excluding participants who had developed incident dementia in the first 2 years of follow-up. However, the association between smoking status and dementia did not change. Then, in the sensitivity analysis excluding participants who had shown poorer cognitive function at the baseline, the association did not change substantially. These findings suggest that the current results are unlikely to have been attributable to reverse causality. With respect to the possible modifying effects of other CRFs on the smoking cessation and incident dementia, we conducted stratification analysis according to a history of stroke or MI, hypertension, and diabetes, respectively. The results suggest that the association between smoking and dementia was independent from other CRFs, indicating that smoking cessation may have a direct relationship to dementia (Table S2). In addition, we also applied the P-spline curve to present the continuous association between years since smoking cessation and dementia after adjustment for multiple covariates (Fig. 3). Among ex-smokers quitting for 3 years or longer, the lower limit of the 95% CI for ln hazard ratio was no longer positive, and further, those quitting for 5 years or longer had a similar level of risk (ln hazard ratio) as never smokers. This continuous association well agreed with our categorical findings.

### Comparisons with previous studies

Our findings were consistent with the results of a recent meta-analysis indicating that, in comparison with never smokers, ex-smokers did not show an increased risk of all-cause dementia (risk ratio 1.01, 95% CI 0.96, 1.06) [13]. To our knowledge, two previous studies have investigated the association between years since smoking cessation and the risk of incident dementia. Choi et al. followed 46,140 Korean men aged 60 years or older for about 8 years and divided ex-smokers into short-term (< 4 years) and long-term (≥ 4 years) quitters [20]. They found that in comparison with current smokers, the risk of dementia decreased among long-term quitters, but not among short-term quitters. Another study from the US also asked about smoking status twice, and found that compared with never smokers, only those who had quit for at least 9 years showed a similar level of incident dementia risk but not current smokers and ex-smokers who had quit for less than 9 years [39]. However, neither revealed a continuous relationship between years since smoking cessation and risk of incident dementia. Our study indicated that the risk for those who had quit for 3 years or longer was reduced to a level comparable with that for never smokers, and thus in comparison with Choi’s study, the effect of smoking cessation upon dementia risk reduction appeared earlier in our study. Additionally, one previous study reported a positive dose–response relationship between years since smoking cessation and cognitive function, that the maximum level of cognitive function can be “regained” after smoking cessation and that the maximum level was reached at about 30 years of age in their population [36].

### Mechanisms

Although the mechanism behind the association of smoking cessation with incident dementia remains unclear, there is evidence that smoking cessation has a direct positive effect on cognitive function, and that this effect appears early after stopping smoking. A 24-month nonrandomized interventional trial has found that temporal declines in Alzheimer’s Disease Assessment Scale-cognitive subscale scores in individuals who stopped smoking for 18 months or more were similar to those among individuals who had never smoked, suggesting that smoking cessation may halt the progression of cognitive decline in a short period [22]. Additionally, our study indicated that the risk of dementia began to decline early after smoking cessation, and that the risk in those who stopped smoking for 3 years or longer was comparable to that in never smokers. On the other hand, smoking cessation may delay or reverse the pathological process of dementia, for example, smoking cessation appears to contribute to an immediate reduction in CVD risk [17], including that for stroke [14] and coronary heart disease [15], a marked increase in plasma antioxidant concentrations, a substantial improvement in plasma resistance towards oxidative challenge [40] and significant improvement in respiratory symptoms and lung function [41]. Additionally, ex-smokers may have a healthier lifestyle than smokers [42], such as consuming more vegetables and fruits, which may exert protective effects against cognitive decline and dementia [43].

### Limitations and strengths

Our study had some limitations that should be mentioned. First, although the response rate was relatively high (72.9%), we cannot rule out the possibility of selection bias among the current study population. Unfortunately, we have no information regarding non-responders. Second, we excluded people with disability before follow-up, as well as persons with other possible (physical) disabilities who did not have dementia. However, we had no information on the cause of disability for those who already had a disability according to LTCI before follow-up. Third, on the other hand, it is possible that people with mild cognitive impairment but not considered to have a disability were included in the analysis; however, the results did not change substantially when we conducted sensitivity analyses, which suggested that there were relatively few cases of early-stage dementia at baseline, which may be less likely to confound the present findings. Fourth, 6333 participants who did not agree to have their LTCI information reviewed were excluded from the analyses, and we have compared their characteristics with those of the participants who agreed (Table S4). As a result, those who disagreed tended to be female, have less education, be current drinkers, have psychological distress and be free from stroke, hypertension or MI. Fifth, self-reported smoking history may have been underreported, which would probably lead to an underestimation of our results, but this can occur among participants both with and without dementia. Lastly, although we considered numerous confounders, residual confounding, such as socioeconomic status or the amount of alcohol consumption, may still have interfered with our findings. Despite these limitations, our study had some strengths, including the large number of participants, the high rate of follow-up and considerable control of confounding factors. Furthermore, we conducted competing-risk models to capture the possible influence from different competing events, employed sensitivity analyses to eliminate the possibility of reverse causality and presented a continuous association of years since smoking cessation and the incident risk of dementia.

### Implications

Our study has focused on the specific question of how long after quitting the elevated risk of incident dementia would drop, about which little evidence has been published to date. Our findings highlight the fact that quitting in a relative short period, that is, 3 years or longer, may contribute to reducing the risk of incident dementia to the same level as that of never smokers. Accordingly, we suggest that smoking cessation should be given high priority among dementia prevention strategies for the elderly. We also believe that our findings can convey a positive and motivating message for supporting smokers to quit. Especially, the declined risk also was observed among older age groups, which indicates that it is never too late to quit for the purpose of reducing the risk of dementia.

## Conclusion

Our study suggests that the risk of incident dementia among ex-smokers becomes the same as that of never smokers if they maintain abstinence from smoking for at least 3 years.

## Electronic supplementary material

Below is the link to the electronic supplementary material.

Supplementary data Table S1. Association between smoking status and incident dementia in sensitivity analyses. Table S2. Association between smoking status and incident dementia in stratification analyses. Table S3. Association between smoking status and incident dementia according to cumulative smoking pack-years. Table S4. Comparison between participants who disagreed to the LTCI and those who agreed (DOCX 27 kb)
