# Supplementary material for: Smoking cessation and incident dementia in elderly Japanese: the Ohsaki Cohort 2006 Study
Source: Eur J Epidemiol. 2020 Feb 15;35(9):851–60. doi: 10.1007/s10654-020-00612-9 (PMC7525275; doi:10.1007/s10654-020-00612-9)
Supplement: Supplementary file 1 — Table S1. Association between smoking status and incident dementia in sensitivity analyses. Table S2. Association between smoking status and incident dementia in stratification analyses. Table S3. Association between smoking status and incident dementia according to cumulative smoking pack-years. Table S4. Comparison between participants who disagreed to the LTCI and those who agreed (DOCX 27 kb) [file 10654_2020_612_MOESM1_ESM.docx]

| Table S1. Association between smoking status and incident dementia in sensitivity analyses | | | | | |
| --- | --- | --- | --- | --- | --- |
|  | Excluding participants whose dementia was ascertained in the first two years ( n =12,164 ) | |  | Excluding participants with worse cognitive function at baseline ( n =7,688 ) | |
| Smoking status | Model 1ᵅ | Model 2ᵝ |  | Model 1 | Model 2 |
| Never smokers | 1.00 (*ref.*)^γ^ | 1.00 (*ref.*) |  | 1.00 (*ref.*) | 1.00 (*ref.*) |
| Current smokers | 1.47 (1.15, 1.89) | 1.41 (1.09, 1.82) |  | 1.46 (1.04, 2.04) | 1.38 (0.98, 1.95) |
| Ex-smokers (years since smoking cessation) | |  |  |  |  |
| ≤2 | 1.55 (1.02, 2.36) | 1.44 (0.94, 2.20) |  | 1.45 (0.81, 2.62) | 1.34 (0.74, 2.43) |
| 3 to 5 | 1.19 (0.76, 1.87) | 1.11 (0.70, 1.75) |  | 1.09 (0.60, 2.00) | 1.01 (0.55, 1.86) |
| 6 to10 | 0.85 (0.55, 1.31) | 0.80 (0.52, 1.24) |  | 1.09 (0.65, 1.83) | 1.05 (0.62, 1.76) |
| 11 to 15 | 1.35 (0.91, 2.01) | 1.26 (0.84, 1.89) |  | 1.47 (0.87, 2.50) | 1.36 (0.80, 2.31) |
| >15 | 1.00 (0.77, 1.30) | 0.96 (0.74, 1.25) |  | 0.78 (0.54, 1.12) | 0.78 (0.54, 1.13) |
| ᵅModel 1 was adjusted for sex and age (continuous). ᵝModel 2 was fully adjusted for the same covariates as Model 2 in the Table 2. ^γ^Hazard ratios (HRs) and 95% confidence intervals (95%CIs) were calculated by Cox proportional hazards models. | | | | | |
|  |  |  |  |  |  |
|  |  |  |  |  |  |
|  |  |  |  |  |  |
|  |  |  |  |  |  |

| Table S2. Association between smoking status and incident dementia in stratification analyses | | | | | | | | |
| --- | --- | --- | --- | --- | --- | --- | --- | --- |
|  | Sex | |  | Age | |  | Education levelᵝ | |
|  | Male | Female |  | ≤75 years | > 75 years |  | <16 years | ≥16 years |
| Never smokers | 1.00 (*ref.*)ᵅ | 1.00 (*ref.*) |  | 1.00 (*ref.*) | 1.00 (*ref.*) |  | 1.00 (*ref.*) | 1.00 (*ref.*) |
| Current smokers | 1.50 (1.17, 1.94) | 1.28 (0.77, 2.13) |  | 1.49 (1.03, 2.16) | 1.15 (0.87, 1.51) |  | 1.34 (0.96, 1.88) | 1.68 (1.22, 2.30) |
| Ex-smokers (years since smoking cessation) | | |  |  |  |  |  |  |
| ≤2 | 1.46 (0.98, 2.17) | 0.84 (0.21, 3.37) |  | 0.94 (0.46, 1.92) | 1.33 (0.86, 2.06) |  | 1.03 (0.56, 1.90) | 1.56 (0.95, 2.59) |
| 3 to 5 | 1.08 (0.71, 1.66) | 0.78 (0.19, 3.16) |  | 0.84 (0.40, 1.78) | 0.93 (0.58, 1.48) |  | 0.86 (0.45, 1.67) | 1.28 (0.73, 2.23) |
| 6 to10 | 1.05 (0.73, 1.53) | 1.08 (0.44, 2.64) |  | 0.87 (0.45, 1.69) | 0.91 (0.62, 1.35) |  | 1.15 (0.69, 1.93) | 1.00 (0.61, 1.64) |
| 11 to 15 | 1.14 (0.78, 1.68) | 2.28 (0.93, 5.54) |  | 0.61 (0.24, 1.55) | 1.19 (0.82, 1.75) |  | 0.97 (0.54, 1.72) | 1.46 (0.91, 2.36) |
| >15 | 0.96 (0.74, 1.23) | 0.87 (0.40, 1.85) |  | 1.14 (0.71, 1.82) | 0.93 (0.71, 1.21) |  | 0.80 (0.56, 1.14) | 1.15 (0.83, 1.60) |
| *P* for interaction | 0.12 | |  | 0.05 | |  | 0.30 | |
|  | History of CVDs^ᵞ^ | |  | History of hypertension | |  | History of diabetes | |
|  | Yes | No |  | Yes | No |  | Yes | No |
| Never smokers | 1.00 (*ref.*) | 1.00 (*ref.*) |  | 1.00 (*ref.*) | 1.00 (*ref.*) |  | 1.00 (*ref.*) | 1.00 (*ref.*) |
| Current smokers | 1.49 (0.68, 3.27) | 1.43 (1.14, 1.78) |  | 1.55 (1.17, 2.05) | 1.33 (0.95, 1.86) |  | 1.32 (0.75, 2.33) | 1.47 (1.17, 1.86) |
| Ex-smokers (years since smoking cessation) | | |  |  |  |  |  |  |
| ≤2 | 2.34 (0.92, 5.97) | 1.19 (0.79, 1.79) |  | 1.53 (0.94, 2.48) | 1.27 (0.72, 2.26) |  | 1.07 (0.41, 2.80) | 1.48 (0.99, 2.21) |
| 3 to 5 | 0.74 (0.21, 2.57) | 1.05 (0.69, 1.58) |  | 1.26 (0.75, 2.10) | 0.80 (0.44, 1.49) |  | 1.33 (0.57, 3.08) | 0.97 (0.62, 1.52) |
| 6 to10 | 2.01 (0.96, 4.21) | 0.89 (0.60, 1.31) |  | 0.99 (0.63, 1.54) | 1.16 (0.70, 1.92) |  | 0.81 (0.36, 1.83) | 1.11 (0.77, 1.59) |
| 11 to 15 | 0.88 (0.31, 2.47) | 1.30 (0.90, 1.88) |  | 1.18 (0.75, 1.86) | 1.28 (0.75, 2.18) |  | 0.97 (0.42, 2.23) | 1.24 (0.84, 1.81) |
| >15 | 1.41 (0.73, 2.73) | 0.87 (0.68, 1.11) |  | 0.89 (0.66, 1.19) | 0.98 (0.68, 1.41) |  | 0.68 (0.39, 1.21) | 0.98 (0.76, 1.25) |
| *P* for interaction | 0.86 | |  | 0.06 | |  | 0.66 | |
| ᵅHazard ratios (HRs) and 95% confidence intervals (95%CIs) were calculated by Cox proportional hazards models adjusted for the same covariates as Model 2 in the Table 2. ᵝAge at completion of education  ^ᵞ^CVDs: cardiovascular diseases, i.e. stroke and myocardial infarction | | | | | | | | |
|  |  |  |  |  |  |  |  |  |
|  |  |  |  |  |  |  |  |  |
|  |  |  |  |  |  |  |  |  |
|  |  |  |  |  |  |  |  |  |
|  |  |  |  |  |  |  |  |  |

| Table S3. Association between smoking status and incident dementia according to cumulative smoking pack-yearsᵅ | | | |
| --- | --- | --- | --- |
|  | Cumulative smoking pack-years | | |
|  | ≤ 20 |  | > 20 |
| Never smokers | 1.00 (*ref.*)^β^ |  | 1.00 (*ref.*) |
| Current smokers | 1.69 (1.16, 2.47) |  | 1.43 (1.12, 183) |
| Ex-smokers (years since smoking cessation) | |  |  |
| ≤2 | 2.03 (0.90 ,4.56) |  | 1.26 (0.81, 1.97) |
| 3 to 5 | 1.00 (0.37, 2.72) |  | 1.16 (0.74, 1.82) |
| 6 to10 | 1.25 (0.47, 3.35) |  | 1.13 (0.77, 1.66) |
| 11 to 15 | 1.75 (0.86, 3.55) |  | 1.13 (0.73, 1.75) |
| >15 | 0.97 (0.69, 1.37) |  | 0.96 (0.72, 1.28) |
| ᵅHazard ratio (HR) and 95% confidence interval (95%CI) were calculated by Cox proportional hazards models. ^β^Multivariable model was fully adjusted for the same covariates as Model 2 in the Table 2. | | | |
|  |  |  |  |
|  |  |  |  |
|  |  |  |  |
|  |  |  |  |

| Table S4. Baseline characteristics according to whether or not agreed to a review to their LTCI information | | | | | |
| --- | --- | --- | --- | --- | --- |
|  | Participants who disagree to a review to their LTCI information |  | Participants who agree to a review to their LTCI information |  | *P* value^α^ |
|  | (n = 6,333) |  | (n= 16,758) |  |  |
| Age, yr (mean±SD) | 74.9±6.5 |  | 74.9±6.6 |  | 0.756 |
| Male, % | 37.1 |  | 43.3 |  | <.0001 |
| Obesity, %^β^ | 31.1 |  | 29.8 |  | 0.1052 |
| Education level (middle school or lower), %^ᵞ^ | 39.7 |  | 31.6 |  | <.0001 |
| Current drinkers, % | 55.7 |  | 52.0 |  | <.0001 |
| Time spent walking (≤0.5 h/day), % | 40.3 |  | 40.3 |  | 0.06 |
| Psychological stress, %^δ^ | 7.5 |  | 6.4 |  | 0.01 |
| History of diseases, % |  |  |  |  |  |
| Stroke | 3.7 |  | 4.8 |  | 0.0003 |
| Hypertension | 51.6 |  | 56.4 |  | <.0001 |
| Myocardial infarction | 4.5 |  | 5.5 |  | 0.0018 |
| Diabetes | 11.4 |  | 12.2 |  | 0.1196 |
| LTCI: Long-term care insurance ᵅP values were calculated by using *χ²* test for variables of proportion and *t*-test for continuous variables. ᵝBody mass index ≥ 25 kg/m^2^ ^ᵞ^Age at completion of education < 16 years old ^δ^ Kessler 6-item psychological distress scale score ≥ 13 | | | | | |
|  |  |  |  |  |  |
|  |  |  |  |  |  |
